# Melatonin-mediated alleviation of salinity stress in diverse mango (*Mangifera indica* L.) rootstock genotypes

**DOI:** 10.3389/fpls.2026.1873066

**Published:** 2026-08-26

**Authors:** Ishu Kumari, Jai Prakash, Kanhaiya Singh, Om Prakash Awasthi, Prabhanjan Rane, Shikha Jain, Chaithra T. S., Gulshan Kumar, Vinay Kumar, Ajay K. N., Bindu Praveena, Sudhir Kumar, Ram Asrey, Dharmendra Singh, Ranjeet Ranjan Kumar

**Affiliations:** 1 Division of Fruits and Horticultural Technology, ICAR-Indian Agricultural Research Institute (IARI), New Delhi, India; 2 Division of Plant Physiology, ICAR-Indian Agricultural Research Institute (IARI), New Delhi, India; 3 Division of Food Science and Post-Harvest Technology, ICAR-Indian Agricultural Research Institute (IARI), New Delhi, India; 4 Division of Genetics, ICAR-Indian Agricultural Research Institute (IARI), New Delhi, India; 5 Division of Biochemistry, ICAR-Indian Agricultural Research Institute (IARI), New Delhi, India

**Keywords:** antioxidant enzymes, ionic toxicity, mango, melatonin, salinity stress

## Abstract

Mango (*Mangifera indica* L.), a tropical fruit crop with significant commercial value, is particularly vulnerable to salt stress. Salinity exposure causes severe physiological disruptions, including leaf burning, chlorosis, and stunted growth, especially in the early phases of plant development, and can be mitigated by salt tolerance. The current study evaluated the effectiveness of exogenous melatonin in mitigating salinity stress at 60 mM NaCl. The findings showed that applying melatonin at an optimal level of 150 μM improved photosynthetic efficiency, relative water content, and membrane stability index, thereby enhancing plant performance. Furthermore, melatonin treatment significantly reduced the build-up of malondialdehyde, a marker of membrane lipid peroxidation, while maintaining increased levels of phenol, proline, and total sugars. Increased activity of superoxide dismutase, catalase, peroxidase, and ascorbate peroxidase enzymes, which together reduced oxidative stress, indicated a stronger antioxidant defense system, strongly linked to enhanced tolerance. Correlation analysis further revealed strong positive relationships among physiological and biochemical parameters, whereas malondialdehyde exhibited negative correlations with all other traits. Also, principal component analysis of six rootstock genotypes under salinity showed that melatonin enhanced stress tolerance in Kurukkan, K 106, and Olour by boosting antioxidant enzymes and preserving photosynthetic pigments. Overall, our findings demonstrate that melatonin increases mango rootstock resilience in saline environments by preserving cellular stability and regulating redox equilibrium.

## 1 Introduction

Mango (*Mangifera indica* L.), a prominent member of the Anacardiaceae family, stands as one of the most prominent tropical and subtropical fruit crops globally. Renowned for its exceptional organoleptic properties and substantial economic value, it currently ranks as the fifth most consumed fruit globally after citrus, banana, grape, and apple, and the second most popular tropical fruit (Halder et al., 2024; Neguse et al., 2019). Its rich nutritional profile, distinctive flavor, pleasant aroma, and notable health benefits have earned it the title of the “king of fruits.” In India, mango cultivation covers about 2.42 million hectares, producing 23.44 million tons in 2024-25 (DA&FW, 2025). Despite its global and national importance, mango cultivation is increasingly challenged under changing climatic conditions, which expose the crop to a wide range of biotic and abiotic stresses that adversely affect growth, productivity, and fruit quality. Among these, soil salinity has emerged as a major constraint, particularly in arid and semi-arid regions, where intensive irrigation accelerates salt accumulation in the rhizosphere. Worldwide, over 900 million hectares of land are affected by excessive salt buildup (Yang et al., 2026). In India, approximately 6.74 million hectares of land are currently affected by soil salinization, with the affected area expanding at an estimated annual rate of nearly 10%. Projections indicate that, if this trend continues, up to 50% of arable land could be affected by salinity by the mid-21st century (Kumar and Sharma, 2020).

Salinity affects plant physiology via ionic toxicity and osmotic stress, triggering oxidative bursts. Excessive reactive oxygen species (ROS) induce lipid peroxidation and structural damage, severely impairing membrane integrity, photosynthetic output, and metabolic functions (Nawaz et al., 2023). Mango trees are inherently salt-sensitive, and exposure to saline environments often results in diminished vegetative vigor, poor fruit quality, and, in extreme cases, tree death (Mahouachi, 2018; Elgendy et al., 2024). In response to these challenges, considerable efforts have been directed toward identifying and utilizing salt-tolerant rootstocks. In mango, polyembryonic rootstocks are particularly valuable because they often exhibit greater uniformity and have been widely used as clonal rootstocks in saline environments. Recent studies have shown that polyembryonic mango genotypes differ significantly in salinity tolerance, supporting their use in rootstock selection for stress-prone areas (Nimbolkar et al., 2023; Perveen et al., 2025). Several polyembryonic mango genotypes, including Bappakkai, Kurukkan, Nekkare, and Olour, have shown promising tolerance to salinity and are increasingly being employed as rootstocks in India (Perveen et al., 2025). However, the variability in tolerance among genotypes necessitates complementary strategies to enhance stress resilience further. One approach that stands out is the exogenous application of plant growth regulators (PGRs). These compounds act as biostimulants by strengthening the plant’s internal defense mechanisms and improving its ability to perceive and respond to environmental stresses through the regulation of hormonal signaling pathways (El-Bially et al., 2022; Hassan et al., 2024). While many osmoprotectants and growth promoters have been studied, melatonin (also known as N-acetyl-5-methoxytryptamine, or MT) has garnered special attention. Originally isolated from bovine pineal glands in 1958 (Lerner et al., 1958), MT was long considered an exclusively animal-based hormone until its fundamental role in plant systems was established (Xie et al., 2022). As a small indole molecule, melatonin plays a key role in regulating diverse physiological and metabolic processes in higher plants (Seth et al., 2023; Kolodziejczyk and Kaźmierczak, 2024). It contributes to stress adaptation and affects several phases of plant growth and development, such as seed germination, vegetative growth, flowering, and fruit production (Seth et al., 2023; Kaya et al., 2019). The strong antioxidant activity of melatonin is one of its most significant characteristics. It reduces oxidative damage by directly scavenging reactive oxygen species and increasing the activity of antioxidant enzymes such as superoxide dismutase (SOD), catalase (CAT), peroxidase (POD), and ascorbate peroxidase (APX) (Zhang et al., 2015; Verma et al., 2026). Furthermore, melatonin promotes the production of compatible solutes such as proline, which helps maintain ionic equilibrium and cellular turgor under stressful conditions, thereby supporting osmotic balance (Singh et al., 2023). It also protects the photosynthetic machinery by preserving the integrity of the thylakoid membrane and preventing pigment degradation (Arnao and Hernández-Ruiz, 2021). Substantial evidence has highlighted the utility of exogenous MT in strengthening the defense mechanisms of various fruit crops like citrus, banana, pomegranate, olive, pistachio etc. against diverse abiotic constraints like heat stress (SKM et al., 2020), salinity (Li et al., 2012; Wei et al., 2022; Yang et al., 2025), drought stress (Korkmaz et al., 2022; Liang et al., 2018, 2019; Hojjati et al., 2024), chilling stress (Cao et al., 2018; Liu et al., 2021), etc. Recent studies in fruit crops have demonstrated the protective effects of melatonin under drought stress. For instance, exogenous melatonin application significantly improved growth and yield-related traits in field-grown banana under semi-arid conditions, highlighting its potential to enhance plant performance under water-deficit conditions (Hassan et al., 2022).

While the application of MT in mango (*Mangifera indica* L.) has been explored in the context of biotic pressures like anthracnose (Xiang et al., 2025; Lu et al., 2025) and gall midge (Sinha et al., 2025), as well as abiotic factors such as chilling injury (Xu et al., 2023; Bhardwaj et al., 2022; Kebbeh et al., 2023) and moisture stress (Trivedi et al., 2024), its role in alleviating salinity stress remains largely unaddressed. Consequently, the present study seeks to systematically elucidate the mechanisms by which exogenous MT mitigates salt-induced toxicity across distinct mango rootstock genotypes with varying tolerance levels, and to determine the optimal application dose through physiological and biochemical assessments. The findings are expected to provide practical insights for enhancing mango cultivation in salt-affected regions through the strategic application of MT.

## 2 Materials and methods

### 2.1 Experimental design

The experiment was conducted in a controlled glasshouse (31/26 ± 5 °C day/night temperature, approx. 60% relative humidity, photoperiod of 10–12 hr., light intensity ranging from 25600 to 54000 lux) at the ICAR-IARI, New Delhi, located at 28°35’ North, 77°12’ East in the year 2025. Both salt-tolerant and salt-susceptible genotypes were employed to evaluate the effects of melatonin under salinity stress. Six mango (*Mangifera indica* L.) rootstock genotypes: Kurukkan, Turpentine, K106, K109, H 07 and Olour were used as experimental material. Fruits were collected, and the extracted stones were sown in pots (top diameter of 29 cm, bottom diameter of 18.5 cm, a height of 35 cm) in July to raise seedlings. Each pot was filled with a mixture of sandy loam soil and farmyard manure in a 4:1 (v/v) ratio. Irrigation was applied to maintain the soil at field capacity throughout the growth period. Half-strength Hoagland’s nutrient solution was applied thrice during this period. After six months, uniform and vigorous seedlings were selected for the study. The experiment followed a factorial arrangement in a Completely Randomized Design (CRD) with three replications, and each replication contained four seedlings. The treatments consisted of: (i) T_0_ -Without NaCl (Control), (ii) T_1–_60 mM NaCl, (iii) T_2–_60 mM NaCl + 150 μM MT, and (iv) T_3–_60 mM NaCl + 200 μM MT.

Salinity stress was gradually imposed on all NaCl-treated pots through soil drenching with a 60 mM NaCl solution. Leachate collected in the trays was returned to the respective pots to ensure uniform salt exposure. Before the separation of experimental groups, these pots received four NaCl applications at 4-day intervals over a period of 16 days. After the fourth application, plants were allocated to their respective treatment groups, and salinity applications continued every four days for an additional 24 days. The total duration of the experiment was 40 days. The control group received tap water with a pH of 6.8 and an EC of 0.27 dS m^-1^.

**Figure 1:**
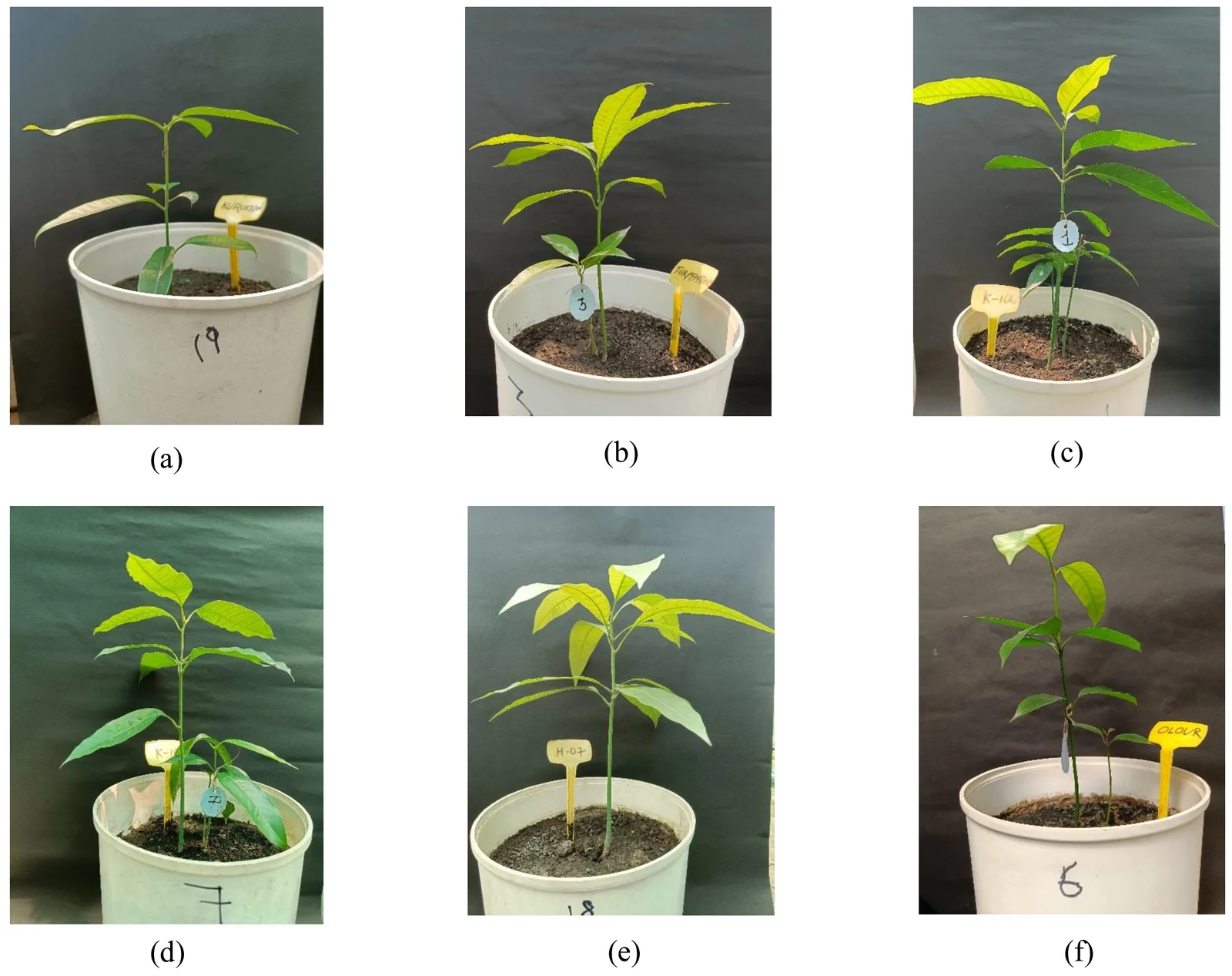
Different mango rootstock genotypes: **(a)** Kurukkan, **(b)** Turpentine, **(c)** K 106, **(d)** K 109, **(e)** H 07, and **(f)** Olour, without NaCl treatment.

**Figure 2:**
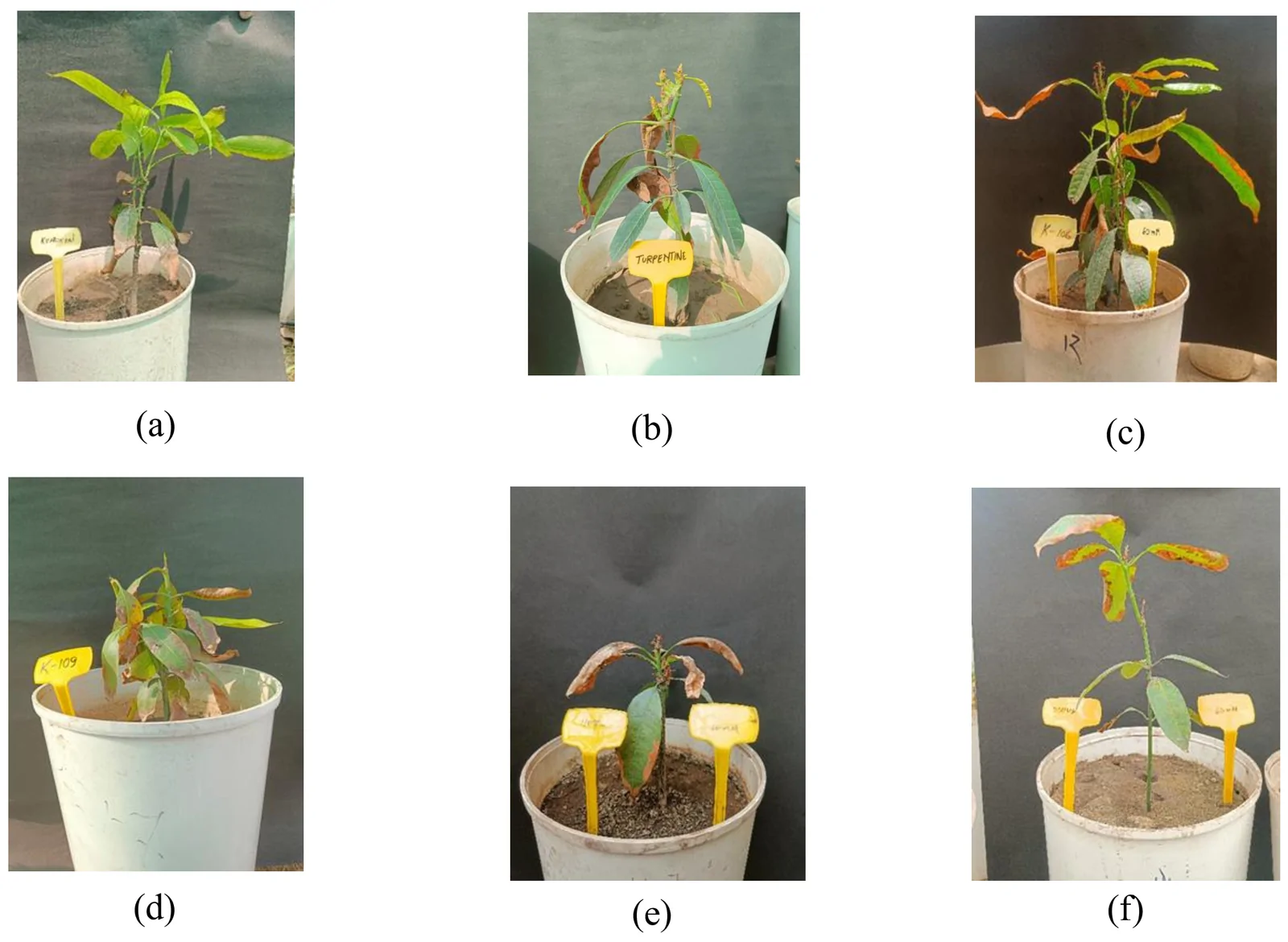
Different mango rootstock genotypes: **(a)** Kurukkan, **(b)** Turpentine, **(c)** K 106, **(d)** K 109, **(e)** H 07, and **(f)** Olour, under 60 mM NaCl. The salinity was imposed gradually at 4-day interval for 40 days.

**Figure 3:**
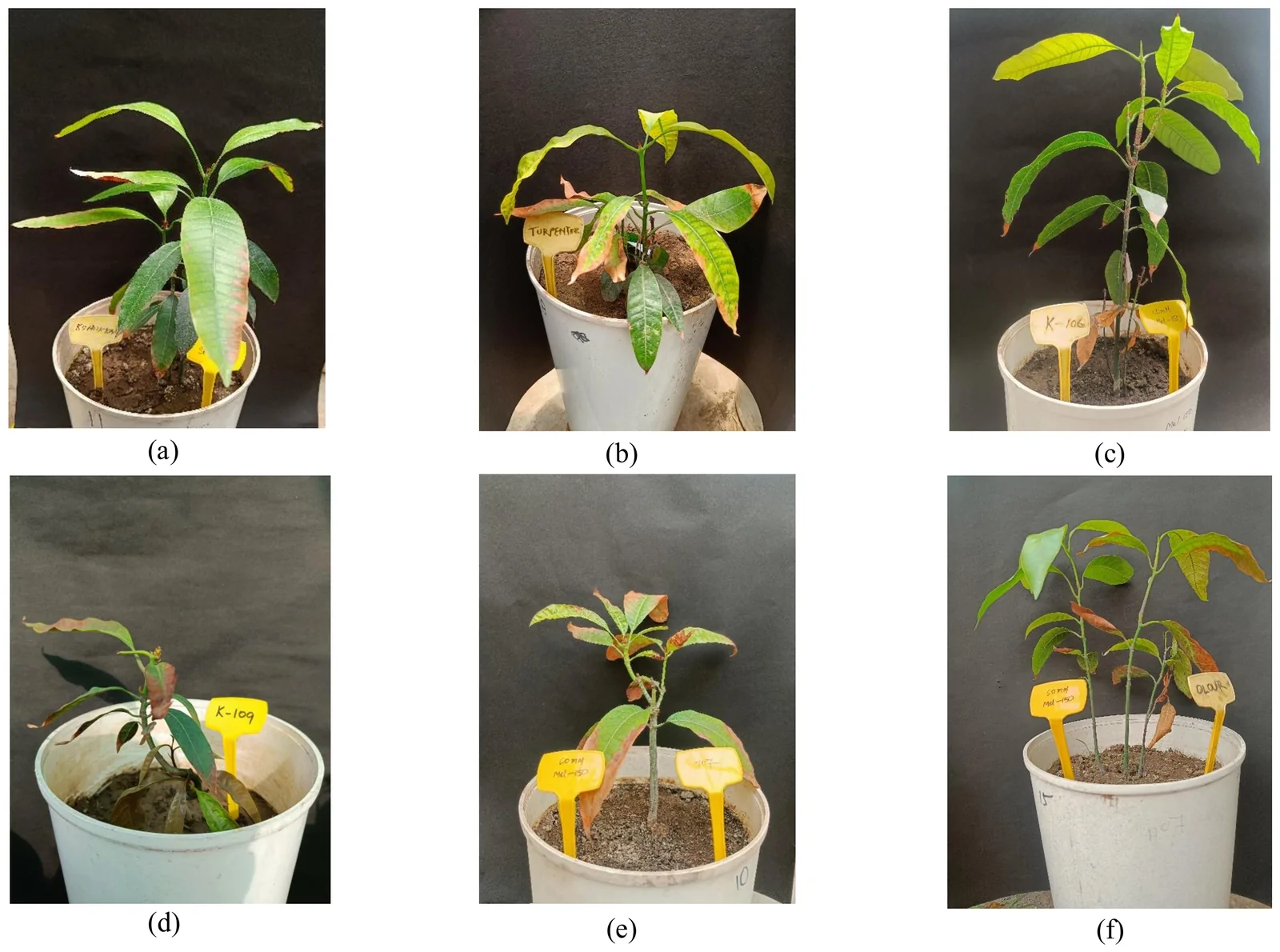
Different mango rootstock genotypes: **(a)** Kurukkan, **(b)** Turpentine, **(c)** K 106, **(d)** K 109, **(e)** H 07, and **(f)** Olour, under 60 mM NaCl + 150 μM MT treatment. The salinity was imposed at 4-day interval for 40 days, following the fourth salt application melatonin treatments were administered via foliar application at weekly intervals for a total of four applications (25 ml MT solution per treatment).

**Figure 4:**
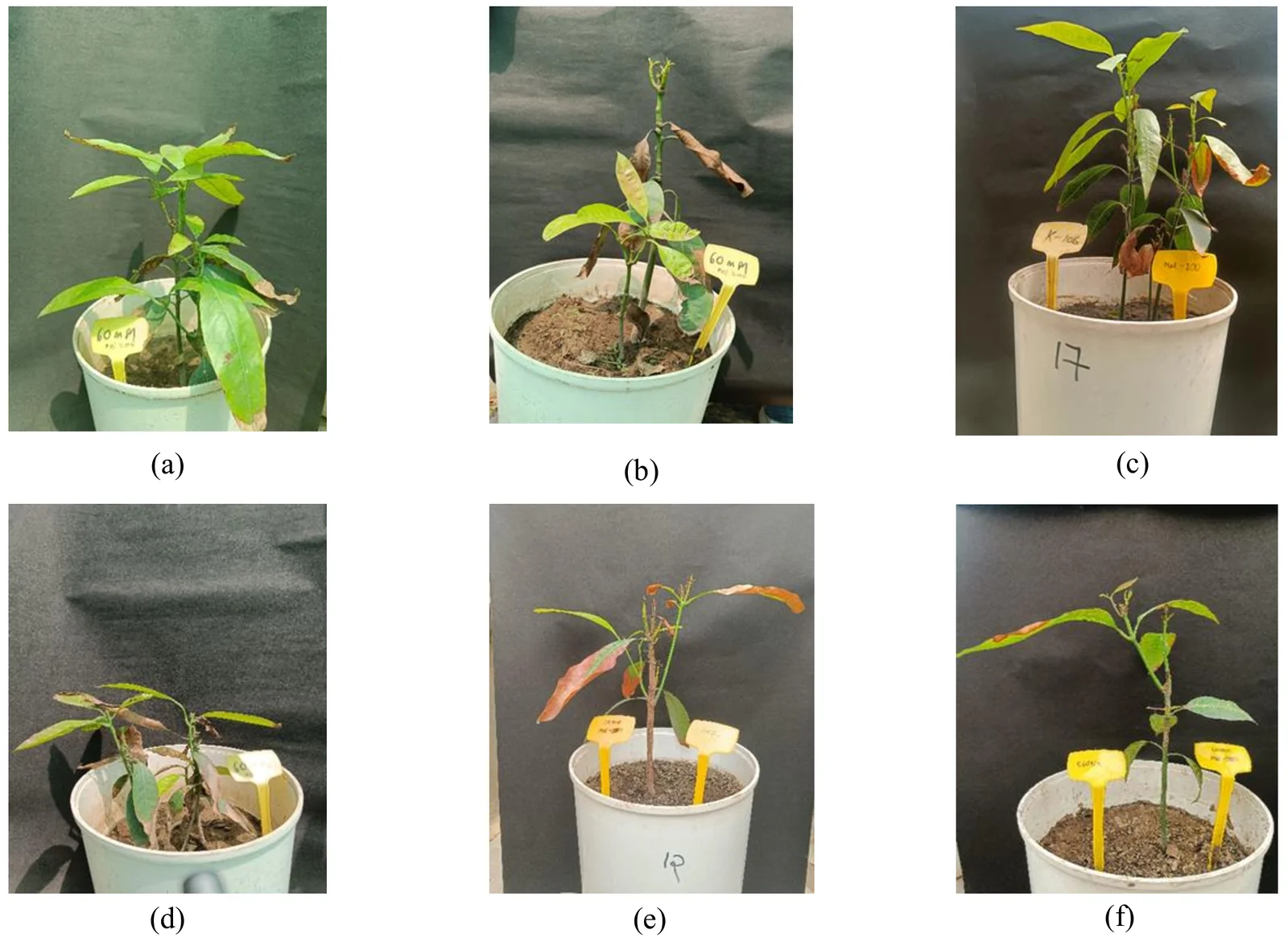
Different mango rootstock genotypes: **(a)** Kurukkan, **(b)** Turpentine, **(c)** K 106, **(d)** K 109, **(e)** H 07, and **(f)** Olour, under 60 mM NaCl + 200 μM MT treatment. The salinity was imposed at 4-day interval for 40 days, following the fourth salt application melatonin treatments were administered via foliar application at weekly intervals for a total of four applications (25 ml MT solution per treatment).

Following the establishment of treatment groups, control and NaCl-treated (without melatonin application) plants (**Figures 1**, **2**) were sprayed with distilled water to ensure uniformity in application. In contrast, treatment groups T_3_ and T_4_ received foliar applications of MT at concentrations of 150 μM and 200 μM, respectively. Melatonin was applied at weekly intervals, with a total of four applications. Each plant received 25 mL of the respective treatment solution per application. A stock solution of Melatonin (Sigma-Aldrich Chemicals Pvt. Ltd., India) was prepared by dissolving it in a small amount of 99.9% ethanol. The volume was made up with double-distilled water, and the solution was stored at -20 °C to maintain stability. Working solutions at 150 μM and 200 μM were prepared by appropriate dilution of the stock solution. The treatments were administered four times at weekly intervals (**Figures 3**, **4**). Care was taken to maintain adequate spacing between treatment sets during application to avoid cross-contamination and preserve the accuracy of treatment effects.

### 2.2 Sampling procedure

One week after the final melatonin (MT) application, fully expanded, mature leaves (third to fifth from the apex) were selected to ensure developmental uniformity across all treatments (Liang et al., 2019; Hassan et al., 2022). Leaves exhibiting complete necrosis were excluded, as they are physiologically inactive; however, partially affected leaves were included where appropriate. All samples were collected following the same criteria across treatments and genotypes to minimize sampling bias.

### 2.3 Physiological characterization

#### 2.3.1 Determination of RWC and MSI

Relative Water Content (RWC) was measured from leaf samples using the following formula, as suggested by Pieczynski et al. (2013).


RWC (%) = 100 x [(Fresh Weight – Dry Weight)/(Turgid Weight – Dry Weight)]

Membrane Stability Index (MSI) was calculated using the Dwivedi et al. (2018) method from 200 mg of fresh leaf tissue. After 30 minutes of incubation in distilled water at 40 °C, the samples’ initial electrical conductivity (EC_1_) was measured using ECTestr 11+ (Thermo Fisher Sci.). The final electrical conductivity (EC_2_) was then measured after the same samples were incubated at 100 °C for 10 minutes. The following formula was used to determine MSI:


$$\text{MSI}=\;\left[1-\;\left(\text{E}{\text{C}}_{1}/\text{E}{\text{C}}_{2}\right)\right]\text{ x }100$$

#### 2.3.2 Determination of leaf gas exchange parameters

Gas exchange parameters were recorded using the LCi-SD portable photosynthetic meter (ADC BioScientific Ltd.). The variables measured included net photosynthetic rate (A), transpiration rate (E), stomatal conductance (gs), and intercellular CO_2_ concentration (Ci).

#### 2.3.3 Determination of photosynthetic pigments and salt damage index

After weighing 100 mg of fresh tissue, it was added to 10 ml of analytical-grade dimethyl sulfoxide (DMSO). The samples were allowed to cool to room temperature after incubation for 4 hours at 65 °C in a water bath (PID-75D, Thermotech) to ensure complete pigment extraction. A UV–19001 (SHIMADZU) double-beam spectrophotometer was used to measure absorbance at 645, 663, and 480 nm, with DMSO serving as the blank. Chlorophyll a (Chl a), chlorophyll b (Chl b), total chlorophyll (Total Chl), chlorophyll a/b ratio, and carotenoid content were calculated using the formulas suggested by Arnon (1949), and pigment concentrations were represented on a fresh weight basis:


$$\text{Chlorophyll a }\left(\text{mg }{\text{g}}^{-1}\right)\;=\;\left[\left(12.7\text{ A}663\;–\;2.69\text{ A}645\right)\text{ x V}\right]/\left(1000\text{ x W}\right)$$

$$\text{Chlorophyll b }\left(\text{mg }{\text{g}}^{-1}\right)\;=\;\left[\left(22.9\text{ A}645\;–\;4.68\text{ A}663\right)\text{ x V}\right]/\left(1000\text{ x W}\right)$$

$$\text{Total chlorophyll }\left(\text{mg }{\text{g}}^{-1}\right)\;=\;\left[\left(8.02\text{ A}663\right)\;+\;\left(20.2\;\text{A }645\right)\right]\text{ x V}/\left(1000\text{ x W}\right)$$

Chl a/b = Chl a/Chl b

$$\text{Carotenoid }\left(\text{mg }{\text{g}}^{-1}\right)\;=\;[\text{A}480\;+\left(0.114\left(\text{A}663\right)\;-\;\left(0.638-\text{A}645\right)\right]\;\times \text{ V}/1000\;\times \text{ W}$$

Where A = Absorbance at the given wavelengths 645 nm, 663 nm, and 480 nm; V =Final volume of DMSO in mL.

The salt damage index (SDI) of the seedlings was evaluated based on overall plant health and the extent of visible leaf injury, following the method described by Shankar et al. (2023). Damage severity was classified into four categories: Level 1 (0–1), no damage; Level 2 (1–2), moderate damage; Level 3 (2–3), severe damage; and Level 4 (3–4), extreme damage. The SDI was subsequently calculated using the formula given below:

SDI = (level value x no. of leaves)/total no. of leaves

### 2.4 Biochemical characterization

#### 2.4.1 Determination of antioxidant enzyme activity

One gram of freshly collected leaf samples was ground using a mortar and pestle and extracted with 10 ml of 0.1 M potassium phosphate buffer (pH 7.5). 0.5 mM EDTA was added to the buffer for SOD, CAT, and POD analysis, whereas the APX extraction additionally contained 1 mM ascorbic acid. The HERMLE Z 323K refrigerated centrifuge was used to centrifuge the homogenate at 12,000 rpm for 20 minutes at 4 °C. The resulting clear supernatant was utilized for enzyme tests. Using the Dhindsa et al. (1981) procedure, SOD activity was assessed based on its capacity to prevent superoxide radicals from reducing nitro blue tetrazolium (NBT). A UV–19001 (SHIMADZU) double-beam spectrophotometer was used to measure SOD activity at 560 nm, defined as the enzyme quantity required to produce 50% suppression of NBT reduction. According to Aebi (1984), CAT activity was measured by monitoring the decrease in H_2_O_2_ absorbance at 240 nm (UV–19001, SHIMADZU), and enzyme activity was calculated as the rate of H2O_2_ breakdown over 1 min. The increase in absorbance at 470 nm (UV–19001, SHIMADZU) caused by the oxidation of guaiacol to tetraguaiacol was used to measure POD activity (Castillo et al., 1984). The reaction mixture (final volume 3 mL) consisted of 16 mM guaiacol, 2 mM H2O_2_, 50 mM phosphate buffer (pH 6.1), and 0.1 mL of the enzyme extract. Tetraguaiacol’s extinction coefficient (ϵ = 26.6 mM^-1^ cm^-1^) was used to calculate the enzyme activity. APX activity was calculated from the drop in absorbance at 290 nm (UV–19001, SHIMADZU) induced by ascorbate oxidation (Nakano and Asada, 1981). After adding 0.2 ml H_2_O_2_ to start the reaction, the absorbance drop was recorded for 30 seconds.

#### 2.4.2 Determination of osmomodulating substances

Total sugars (TS) were estimated using the anthrone method (Dubois et al., 1956). Fresh tissue (100 mg) was extracted in 10 ml of 50% ethanol and centrifuged at 4000 rpm for 15 min using HERMLE Z 323K refrigerated centrifuge. For analysis, the resultant supernatant was obtained. 200 mg of anthrone was dissolved in 100 mL of concentrated H_2_SO_4_ to create the anthrone reagent. In a water bath (PID-75D, Thermotech), an aliquot (0.1 ml) of the extract was first evaporated to dryness, then redissolved and treated with 4 ml of freshly prepared anthrone reagent. The combination was heated for 10 minutes in a boiling water bath (PID-75D, Thermotech), cooled to room temperature, and absorbance was measured at 620 nm (UV–19001, SHIMADZU) to quantify the sugar content.

The method of Bates et al. (1973) was slightly modified to determine the proline content (P) in fresh leaves. A pre-chilled mortar and pestle were used to homogenize 0.5 g of leaf tissue in 5 ml of 3% sulphosalicylic acid. The clear supernatant was collected for examination after the homogenate was centrifuged at 10,000 rpm for 10 minutes using a HERMLE Z 323K chilled centrifuge. In test tubes, an aliquot of 2 ml supernatant was combined with sulphosalicylic acid, glacial acetic acid, and freshly made acid-ninhydrin reagent. After incubating the reaction mixture for one hour at 100 °C in a boiling water bath (PID-75D, Thermotech), the tubes were placed in an ice bath to stop the process. Four milliliters of toluene were then added, followed by thorough mixing. Four milliliters of toluene were then added, and everything was thoroughly mixed. A spectrophotometer (UV–19001, SHIMADZU) was used to measure the absorbance of the upper toluene phase at 528 nm after thorough separation and room-temperature equilibration. Fresh weight was used to calculate and express the proline content.

#### 2.4.3 Determination of total phenol and malondialdehyde

Total phenol (TP) was assessed using the Folin–Ciocalteu method (Singleton and Rossi, 1965) with slight modifications. Approximately 100 mg of frozen leaf tissue was crushed in 5 mL of 80% ethanol, and the mixture was centrifuged using the HERMLE Z 323K refrigerated centrifuge for 20 min. at 15,000 rpm. 2.5 ml of ethanol was used to extract the remaining particles further, and the combined extracts were dried by evaporation. Ten milliliters of distilled water were used to reconstitute the dried residue. For analysis, 0.5 ml of the extract (properly diluted) was mixed with 0.5 ml of Folin-Ciocalteu reagent, followed by the addition of 2 ml of 20% sodium carbonate. After a brief 1-minute exposure to 100 °C in a PID-75D Thermotech water bath, the mixture was left to develop color at room temperature for 1 hour. A UV-Vis double-beam spectrophotometer (UV–19001, SHIMADZU) was used to detect absorbance at 750 nm. The phenol concentration was then computed and given as mg/GAE.

Malondialdehyde (MDA) content was estimated using a modified version of the method described by Heath and Packer (1968). After homogenizing fresh leaf tissue (0.5 g) in 10 ml of 0.1% trichloroacetic acid (TCA), it was centrifuged (HERMLE Z 323K) for 15 minutes at 15,000 rpm. After that, 4 ml of 0.5% (w/v) thiobarbituric acid (TBA) prepared in 20% TCA was combined with a 1 ml aliquot of the supernatant. In the Thermotech PID-75D water bath, the reaction mixture was heated at 95 °C for 30 minutes, and then quickly cooled to room temperature. After that, it was centrifuged for 10 minutes at 4 °C at 10,000 rpm. Using a spectrophotometer (UV–19001, SHIMADZU), the absorbance of the clear supernatant was measured at 532 nm and adjusted for non-specific turbidity by deducting the value at 600 nm. The appropriate extinction coefficient (ϵ) was used to compute the MDA concentration, which was then reported as nmol g^-1^ fresh weight.


MDA (nmol/g FW) = [(A532−A600) x Vx 1000/ϵ] x W

where A532 is the absorbance at 532 nm wavelength, A600 is the absorbance at 600 nm wavelength, V is the volume of the crushing medium, W is the fresh weight of the leaf, and ϵ is the specific extinction coefficient (=155 mM/cm).

### 2.5 Principal component analysis

Principal component analysis (PCA) in IBM SPSS Statistics version 25 was used to evaluate the impact of MT on reducing salt stress across different mango rootstock genotypes. Eigenvalues, percentage variance, and cumulative percentage variance were all included. To determine the primary component factors, a scree plot was created using the data. All figures were prepared using Origin Pro (OriginLab Corporation, Northampton, MA, USA).

### 2.6 Statistical analysis

To evaluate the impact of treatments on mango genotypes, two-way ANOVA was used to analyze all of the experimental data. Tukey’s Honest Significant Difference Test was used for *post hoc* comparisons at a significance level of p< 0.05. Origin Pro 2022 was used to create the graphs, while SPSS 28 (IBM Corp, 2021). IBM SPSS Statistics for Windows. Version 28. IBM Corp., Armonk was used for statistical analysis.

## 3 Result

### 3.1 Changes in RWC and MSI in response to melatonin under salinity stress

**Figure 5:**
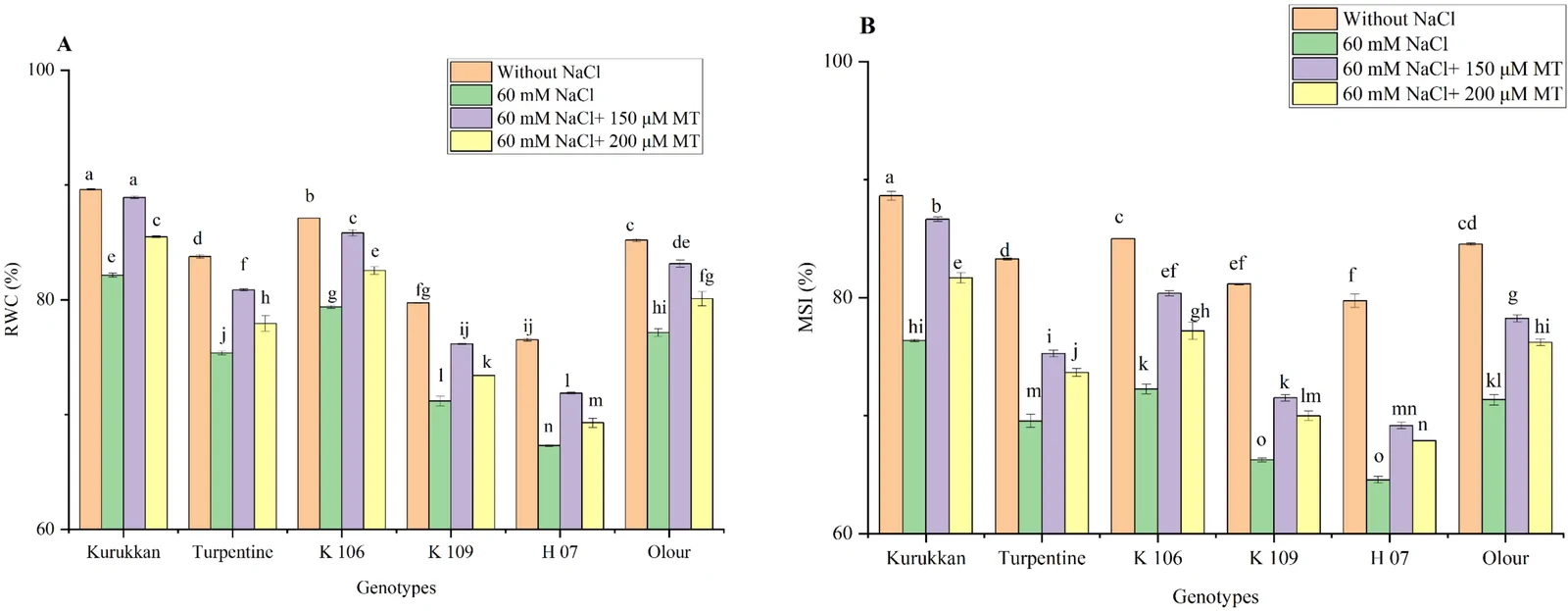
Effect of salinity and melatonin (MT) treatments on **(A)** RWC (relative water content) and **(B)** MSI (membrane stability index) in different mango rootstock genotypes. Six-month-old seedlings of Kurukkan, Turpentine, K106, K109, H 07, and Olour were used as experimental material. Each treatment combination consisted of three replications, with four seedlings per replication (N = 12, where N represents the total number of seedlings per treatment combination). Letters a-o indicated significant differences based on the *Post Hoc*-Tuckey test (p< 0.05), and error bars represent standard error of the mean.

The highest RWC and MSI were recorded in plants maintained under non-saline conditions (Without NaCl) across all mango rootstock genotypes, while plants grown under 60 mM NaCl showed the lowest values. Salinity stress significantly impaired leaf water status and membrane integrity in the investigated mango rootstock genotypes (**Figure 5**). A significant genotype × treatment interaction was evident for RWC (**Figure 5A**), with H 07 under 60 mM NaCl exhibiting the lowest RWC (67.29%) while Kurukkan under non-saline conditions exhibited the highest RWC (89.60%). Similarly, MSI was lowest in H07 under 60 mM NaCl (64.55%), whereas the highest values (88.62%) were recorded in Kurukkan under non-saline conditions (**Figure 5B**).

MT application improved physiological parameters under salinity stress, with a more pronounced effect at 150 µM than at 200 µM. A genotype-specific response was evident, with tolerant rootstocks showing greater improvement than susceptible ones. Under 60 mM NaCl, Kurukkan treated with 150 µM MT exhibited marked increases in RWC (88.92%) as shown in **Figure 5A** and MSI (86.64%) as shown in **Figure 5B**, corresponding to improvements of 8.25% and 13.46%, respectively, over their salt-stressed controls (T_1_). In contrast, 200 µM MT treatment showed comparatively less improvement in the trait, with RWC and MSI values being 85.48% and 81.66%, respectively. A similar trend was observed across all other genotypes. These data suggest that exogenous MT facilitates osmotic adjustment and minimizes electrolyte leakage across a diverse range of mango rootstock genotypes.

### 3.2 Changes in leaf gas exchange parameters in response to melatonin under salinity stress

**Figure 6:**
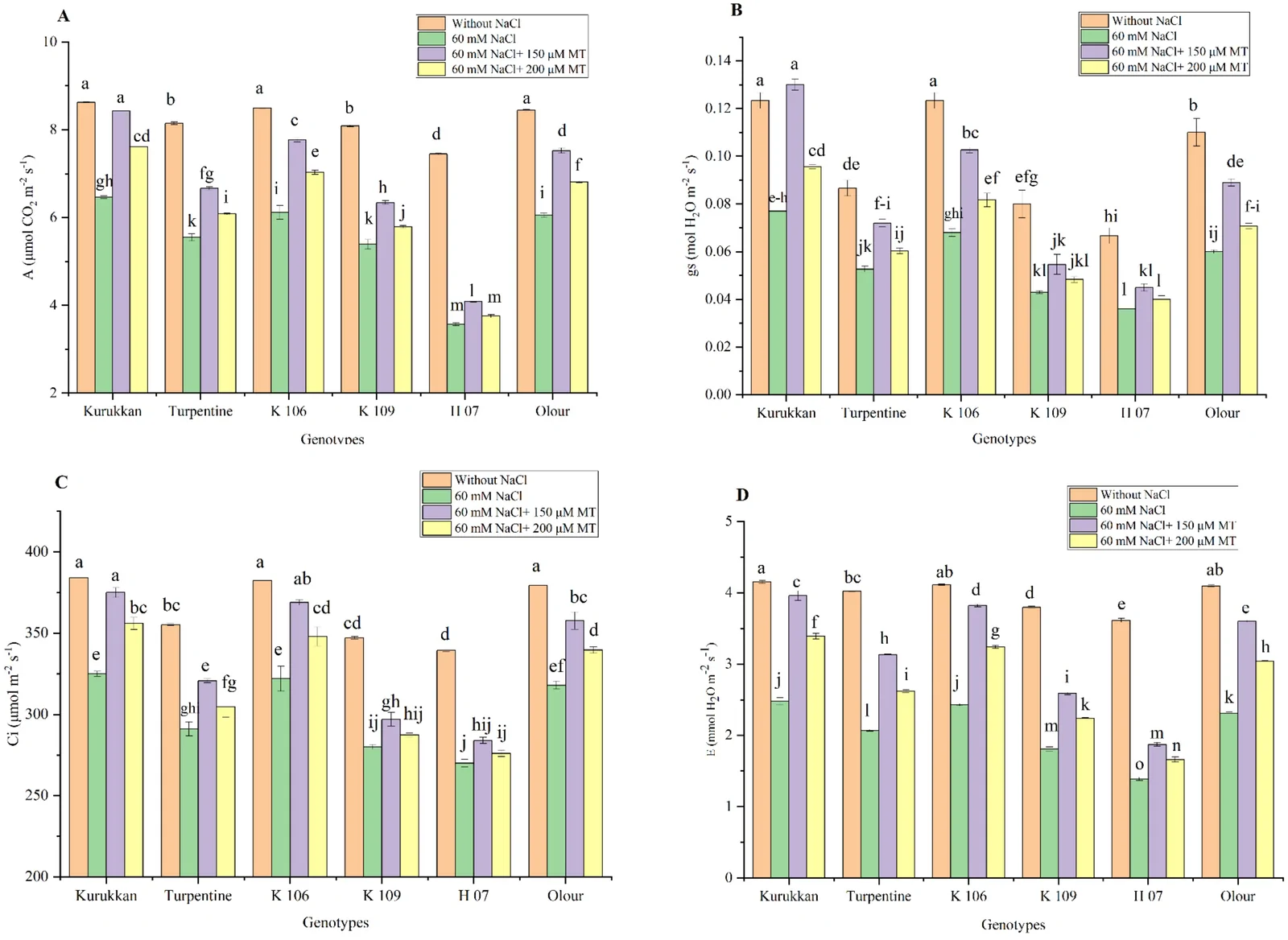
Effect of salinity and melatonin (MT) treatments on **(A)** Photosynthetic rate (A), **(B)** Stomatal conductance (gs), **(C)** Intercellular CO_2_ concentration (Ci), and **(D)** Transpiration rate (E) in different mango rootstock genotypes. Six-month-old seedlings of Kurukkan, Turpentine, K106, K109, H 07, and Olour were used as experimental material. Each treatment combination consisted of three replications, with four seedlings per replication (N = 12, where N represents the total number of seedlings per treatment combination). Letters a-m indicated significant differences based on the *Post Hoc*-Tukey test (p< 0.05), and error bars represent the standard error of the mean.

Gas exchange parameters serve as key indicators for diagnosing limitations to photosynthesis and understanding the balance between transpiration and water-use efficiency under stress conditions. As illustrated in **Figure 6**, gas exchange parameters varied significantly among treatments and genotypes. For photosynthetic rate (A) as shown in **Figure 6A**, the highest value (8.63 μmol m^-2^ s^-1^) was recorded in Kurukkan under non-saline conditions, whereas salinity stress (60 mM NaCl) caused a marked reduction across all genotypes, with the lowest value (3.57 μmol m^-2^ s^-1^) observed in H07. However, exogenous application of MT, particularly at 150 µM, substantially alleviated this reduction. Kurukkan exhibited a better response with a photosynthetic rate of 8.43 μmol m^-2^ s^-1^, whereas H07 showed a comparatively lower value (4.09 μmol m^-2^ s^-1^).

Stomatal conductance (gs), as shown in **Figure 6B**, reached its maximum value (0.13 mol H_2_O m^-2^ s^-1^) in Kurukkan under 60 mM NaCl supplemented with 150 µM MT, which was comparable to the values observed in Kurukkan and K106 under non-saline conditions. The minimum gs value (0.04 mol H_2_O m^-2^ s^-1^) was recorded in H07 under salinity stress as well as under 200 µM MT treatment. Overall, salinity stress significantly reduced gs, however, MT application partially restored stomatal conductance. Kurukkan exhibited higher gs values (0.09 and 0.07 mol H_2_O m^-2^ s^-1^ at 150 µM and 200 µM, respectively), whereas H07 maintained comparatively lower values (0.05 and 0.04 mol H_2_O m^-2^ s^-1^) under the corresponding treatments.

For intercellular CO_2_ concentration (Ci), the highest value (326.83 μmol CO_2_ m^-2^ s^-1^) was observed in Kurukkan under non-saline conditions (**Figure 6C**). Salinity significantly reduced Ci, with H07 recording the lowest (270.00 μmol CO_2_ m^-2^ s^-1^). MT treatment mitigated this reduction, with Kurukkan showing improved Ci (313.08 μmol CO_2_ m^-2^ s^-1^) at 150 µM, whereas H07 recorded a comparatively lower value (255.17 μmol CO_2_ m^-2^ s^-1^).

Transpiration rate (E) also declined under salinity stress (**Figure 6D**). The highest value (4.15 mmol H_2_O m^-2^ s^-1^) was recorded in Kurukkan under control conditions, while the lowest (1.39 mmol H_2_O m^-2^ s^-1^) was observed in H07 under salinity stress. MT application improved E, with Kurukkan recording 2.80 and 2.63 mmol H_2_O m^-2^ s^-1^ at 150 µM and 200 µM, respectively. In contrast, H07 showed lower values of 1.93 and 1.86 mmol H_2_O m^-2^ s^-1^ under the same treatments.

### 3.3 Changes in photosynthetic pigments and salt damage index in response to melatonin under salinity stress

**Figure 7:**
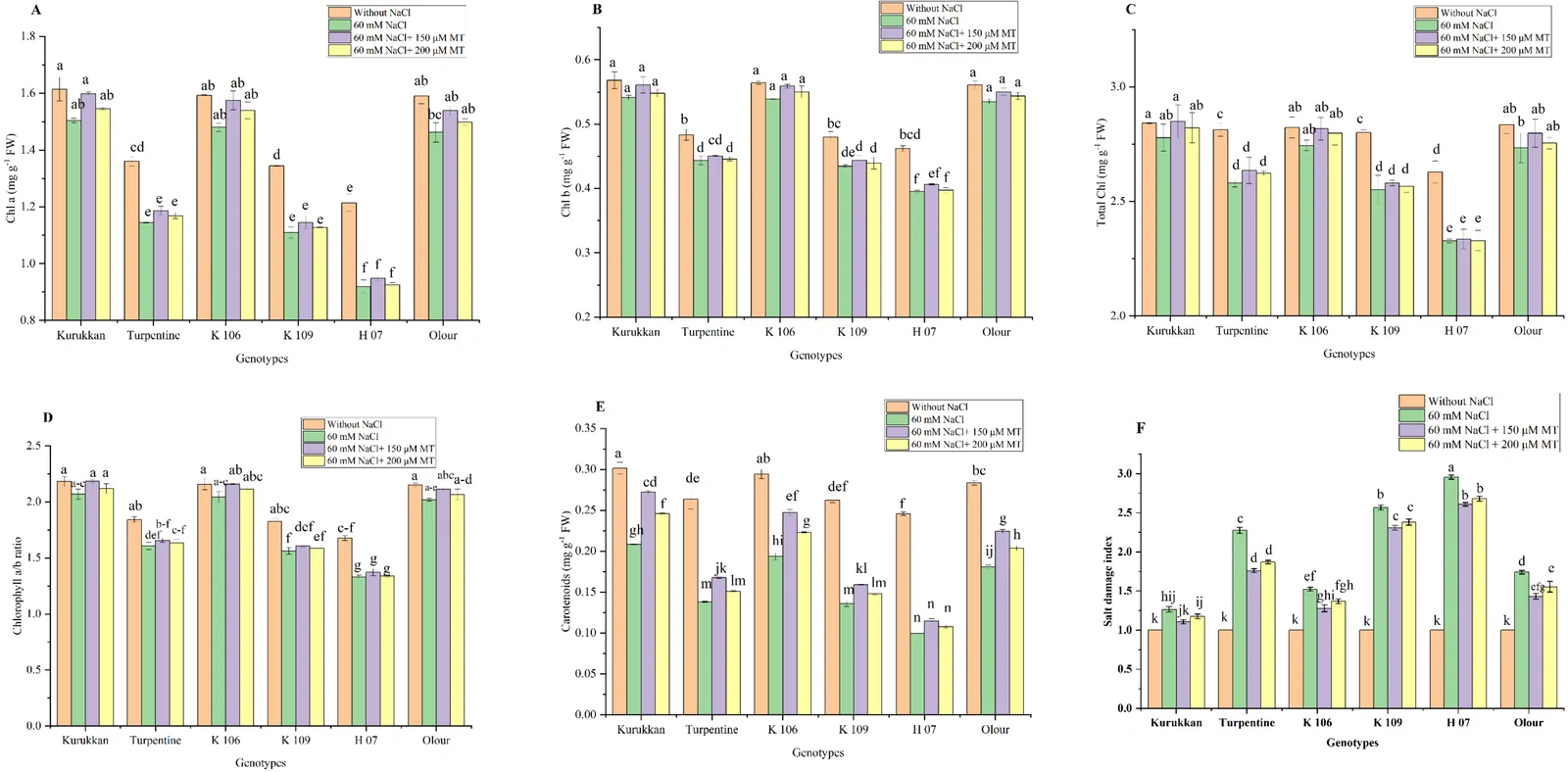
Effect of salinity and melatonin (MT) treatments on **(A)** Chl a, **(B)** Chl b, **(C)** Total Chl, **(D)** Chlorophyll a/b ratio, **(E)** Carotenoids and **(F)** Salt damage index (SDI) in different mango rootstock genotypes. Six-month-old seedlings of Kurukkan, Turpentine, K106, K109, H 07, and Olour were used as experimental material. Each treatment combination consisted of three replications, with four seedlings per replication (N = 12, where N represents the total number of seedlings per treatment combination). Letters a-n indicated significant differences based on the *Post Hoc*- Tuckey test (p< 0.05), and error bars represent the standard error of the mean.

The photosynthetic capacity of the evaluated mango genotypes was notably compromised by salinity, primarily due to accelerated catabolism of photosynthetic pigments. Analysis of the genotype × treatment interaction (**Figure 7A**) revealed that under 60 mM NaCl stress, the H 07 genotype exhibited the highest sensitivity, recording the lowest values of Chl a (0.920 mg g^-1^). The highest Chl a value (1.615 mg g^-1^) was recorded in Kurukkan under non-saline conditions (without NaCl). Exogenous melatonin (MT) application, particularly at 150 µM, significantly improved Chl a content under salinity stress, with Kurukkan showing the maximum recovery (1.599 mg g^-1^), whereas H07 exhibited only a marginal increase (0.949 mg g^-1^). **Figure 7B** illustrates a similar pattern for Chl b, with Kurukkan without NaCl treatment recording the highest value (0.568 mg g^-1^) and H07 under salinity stress displaying the lowest value (0.395 mg g^-1^). MT application at 150 µM enhanced Chl b content in all rootstock genotypes, with significant improvement shown by tolerant genotypes like Kurukkan (0.561 mg g^-1^), while only a slight improvement was noted in H07 (0.406 mg g^-1^). Total chlorophyll content followed the same trend (**Figure 7C**), with the minimum value (1.331 mg g^-1^) recorded in H07 under 60 mM NaCl, while the maximum (2.183 mg g^-1^) was in Kurukkan without NaCl. Application of 150 µM MT resulted in a marked increase of Total Chl in Kurukkan (2.185 mg g^-1^), whereas H07 showed limited recovery (1.372 mg g^-1^). The data for the chlorophyll a/b ratio, as illustrated in **Figure 7D**, show that H07 under salinity stress recorded the lowest value (2.327), indicating an imbalance in pigment composition, while the highest value (2.843) was exhibited by Kurukkan without any NaCl treatment. Melatonin (MT) application further improved this ratio, with Kurukkan exhibiting a higher value (2.849), whereas H07 showed only a marginal increase (2.335).

Carotenoid values, as shown in **Figure 7E**, were reported to be higher in normal control plants (without NaCl), with a maximum value (0.301mg g^-1^) reported in Kurukkan. Salinity stress caused a pronounced reduction in carotenoid content, with the minimum level recorded in H 07 (0.100 mg g^-1^). However, application of MT, particularly at 150 µM, mitigated this reduction and significantly improved carotenoid accumulation under salt stress. The increase in carotenoid content due to 150 µM MT treatment was 30.62% in Kurukkan, 21.38% in Turpentine, 27.88% in K106, 17.11% in K109, 14.94% in H 07, and 24.04% in Olour.

Salt damage index (SDI) values indicated that control plants exhibited no visible damage symptoms (1.0), whereas seedlings exposed to 60 mM NaCl developed varying degrees of necrosis (**Figure 7F**). The most serious damage was recorded in sensitive genotypes such as H07 (2.95), while the lowest was observed in Kurukkan (1.27). MT application mitigated tissue damage and supported plant survival by improving stress tolerance and promoting growth. Relatively tolerant genotypes, including Kurukkan (1.11, 1.18), K106 (1.28, 1.37), and Olour (1.43, 1.56), exhibited lower levels of tissue damage at 150 µM and 200 µM MT, respectively. In contrast, sensitive genotypes such as K109 (2.31, 2.38) and H07 (2.61, 2.68) showed comparatively weaker responses to MT treatment.

### 3.4 Changes in antioxidant enzyme activities in response to melatonin under salinity stress

**Figure 8:**
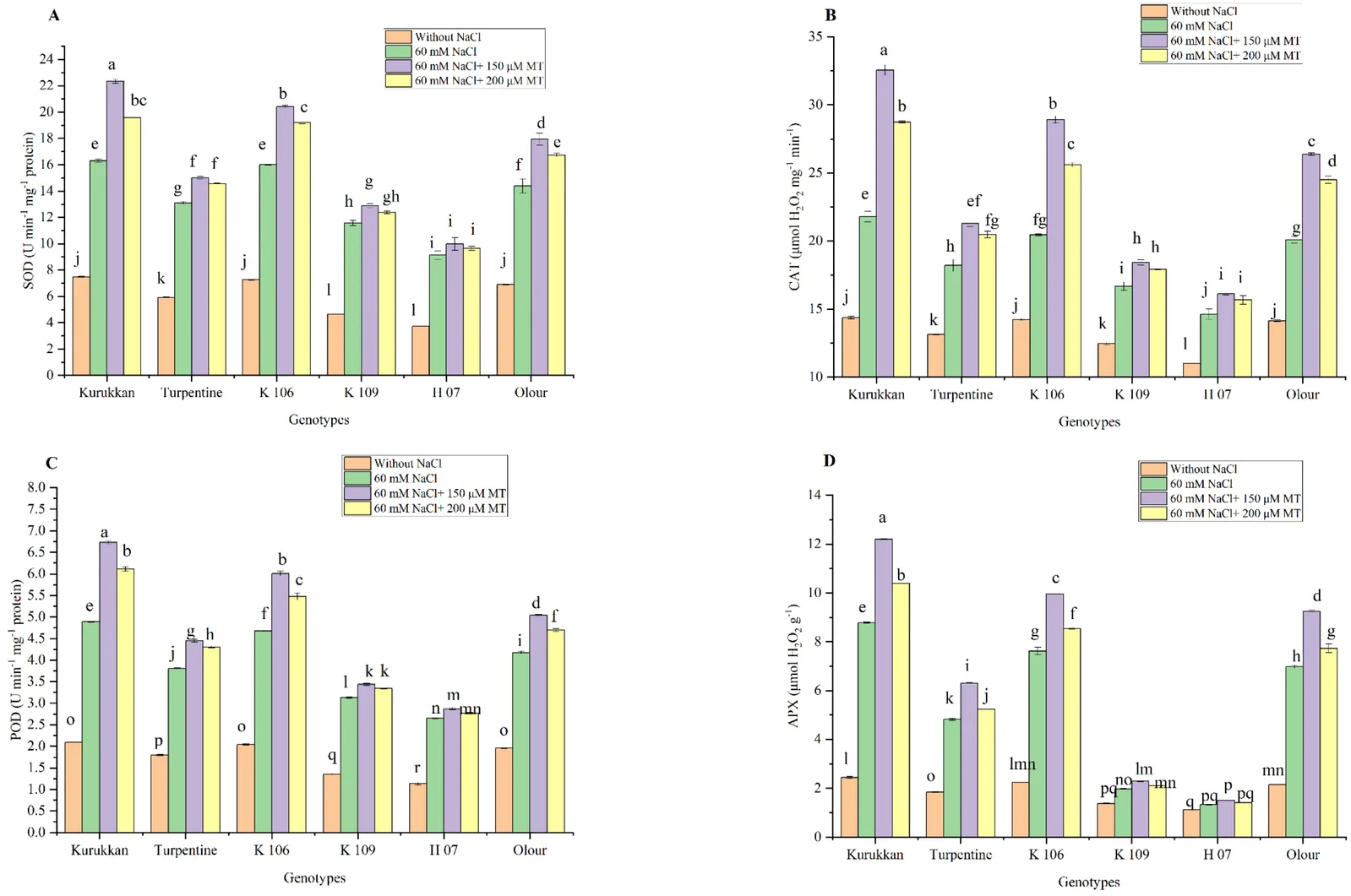
Effect of salinity and melatonin (MT) treatments on **(A)** SOD (superoxide dismutase), **(B)** CAT (catalase), **(C)** POD (peroxidase), and **(D)** APX (ascorbate peroxidase) in different mango rootstock genotypes. Six-month-old seedlings of Kurukkan, Turpentine, K106, K109, H 07, and Olour were used as experimental material. Each treatment combination consisted of three replications, with four seedlings per replication (N = 12, where N represents the total number of seedlings per treatment combination). Letters a-r indicated significant differences based on the *Post Hoc*–Tuckey test (p< 0.05), and error bars represent the standard error of the mean.

Antioxidant enzyme activities were markedly elevated across all mango rootstock genotypes under 60 mM NaCl, and exogenous MT application further enhanced these responses. However, the magnitude of this response varied according to the tolerance capacity of the rootstock genotypes. The highest SOD activity (22.34 U min^-1^ mg^-1^ protein) was recorded in Kurukkan under 60 mM NaCl supplemented with 150 µM MT, whereas the lowest activity (3.73 U min^-1^ mg^-1^ protein) was observed in H07 under non-saline conditions (**Figure 8A**). A similar trend was observed for CAT, with the maximum activity (32.56 μmol H_2_O_2_ mg^-1^ min^-1^) in Kurukkan under 60 mM NaCl with 150 µM MT, while the minimum (11.01 μmol H_2_O_2_ mg^-1^ min^-1^) was recorded in H07 under non-saline conditions (**Figure 8B**). POD activity was also highest (6.73 U min^-1^ mg^-1^ protein) in Kurukkan under 60 mM NaCl treated with 150 µM MT, whereas the lowest value (1.13 U min^-1^ mg^-1^ protein) was noted in H07 under non-saline conditions (**Figure 8C**). APX activity reached its maximum (12.20 μmol H_2_O_2_ g^-1^) in Kurukkan under 60 mM NaCl with 150 µM MT treatment, while the lowest value (1.12 μmol H_2_O_2_ g^-^¹) was observed in H07 under non-saline conditions (**Figure 8D**).

### 3.5 Changes in osmomodulating substances in response to melatonin under salinity stress

**Figure 9:**
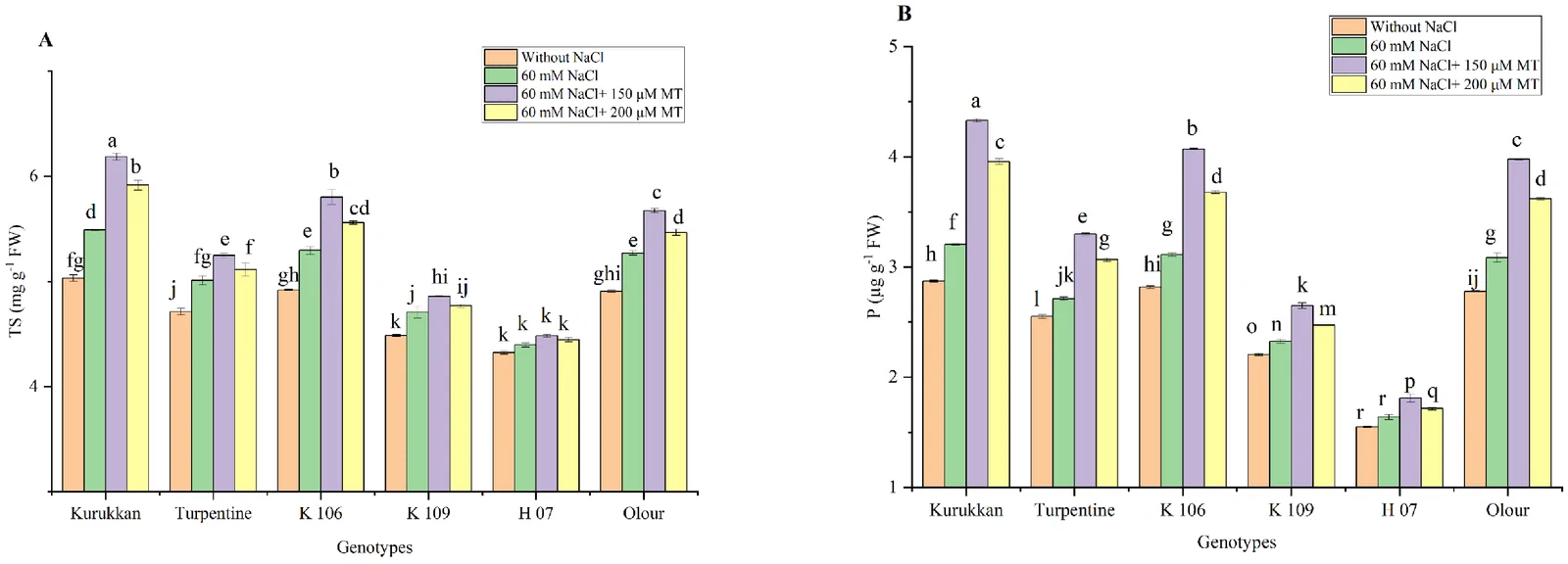
Effect of salinity and melatonin (MT) treatments on **(A)** total sugars (TS) and **(B)** proline (P) in different mango rootstock genotypes. Six month old seedlings of Kurukkan, Turpentine, K106, K109, H 07, and Olour were used as experimental material. Each treatment combination consisted of three replications, with four seedlings per replication (N = 12, where N represents the total number of seedlings per treatment combination). Letters a-q indicated significant differences based on the *Post Hoc*-Tuckey test (p< 0.05), and error bars represent the standard error of the mean.

Accumulation of osmolytes increased markedly in response to salinity stress (60 mM NaCl) across all mango rootstock genotypes, with a further enhancement observed following MT application. For total soluble sugars (**Figure 9A**), the highest concentration (6.19 mg g^-1^ FW) was recorded in Kurukkan under salinity stress supplemented with 150 µM MT. Application of 150 µM MT showed an increment of 12.69%, 9.57%, 7.65%, 4.72%, 3.26%, and 1.97% in Kurukkan, K106, Olour, Turpentine, K109, and H07, respectively, compared to salinity treatment alone.

A similar trend was observed for proline accumulation as illustrated in **Figure 9B**. The maximum proline content (50.55 μg g^-1^ FW) was recorded in Kurukkan seedlings subjected to NaCl stress and treated with 150 µM MT, whereas the minimum value (28.32 μg g^-1^ FW) was noted in H07 under non-saline conditions. MT application significantly enhanced proline levels, with increases of 35.03%, 30.73%, 29.01%, 21.62%, 13.90%, and 10.37% in Kurukkan, K106, Olour, Turpentine, K109, and H07, respectively, relative to salinity-stressed plants without MT.

### 3.6 Changes in total phenol and MDA in response to melatonin under salinity stress

**Figure 10:**
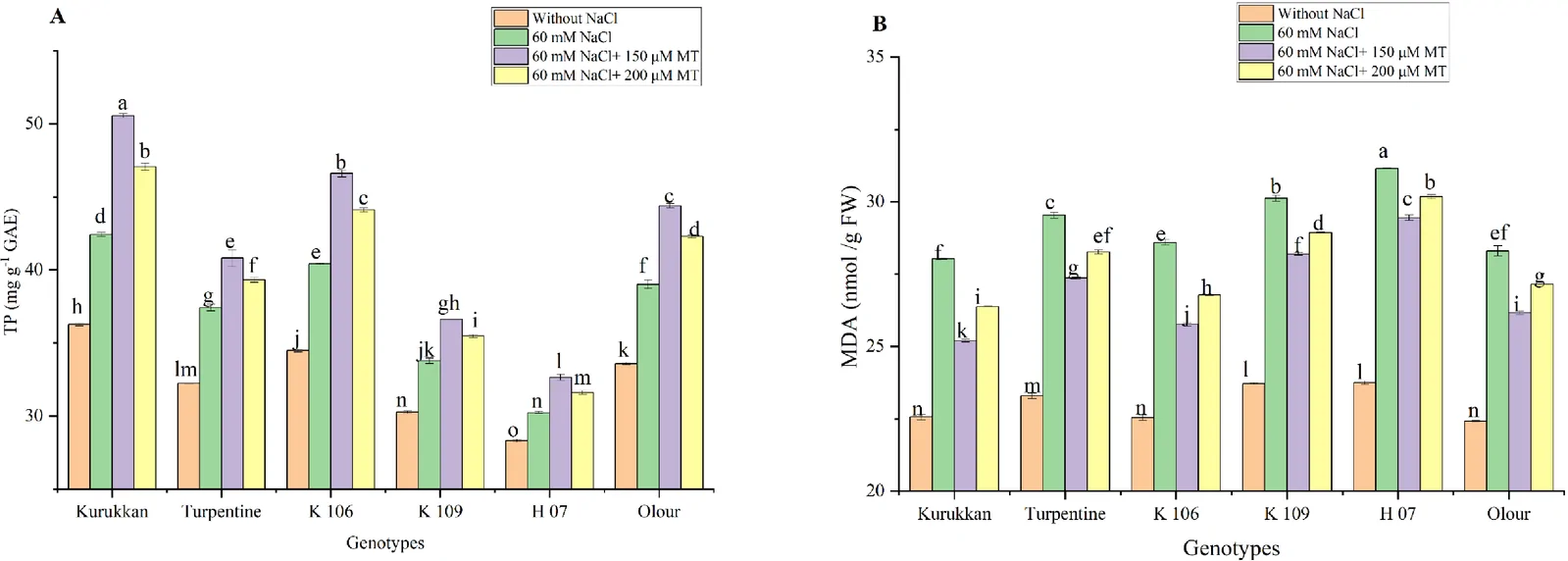
Effect of salinity and melatonin (MT) treatments on **(A)** total phenol (TP) and **(B)** malondialdehyde (MDA) in different mango rootstock genotypes. Six month old seedlings of Kurukkan, Turpentine, K106, K109, H 07, and Olour were used as experimental material. Each treatment combination consisted of three replications, with four seedlings per replication (N = 12, where N represents the total number of seedlings per treatment combination). Letters a-q indicated significant differences based on the *Post Hoc*-Tuckey test (p< 0.05), and error bars represent the standard error of the mean.

Total phenol content varied significantly among treatments and genotypes (**Figure 10A**). The lowest phenol concentration (28.32 mg g^-1^ GAE) was recorded in H07 under non-saline conditions, whereas the highest value (50.55 mg g^-1^ GAE) was observed in Kurukkan under 60 mM NaCl supplemented with 150 µM melatonin (MT). Application of MT enhanced total phenol content across all mango rootstock genotypes. Kurukkan exhibited the highest phenol accumulation, with values of 50.55 mg g^-1^ GAE and 47.06 mg g^-1^ GAE at 150 µM and 200 µM MT, respectively. In contrast, H07 showed comparatively lower phenol levels, recording 32.65 mg g^-1^ GAE and 31.61 mg g^-1^ GAE at the corresponding MT concentrations.

The lowest malondialdehyde (MDA) content was recorded in Kurukkan (22.42 nmol g^-1^ FW) under non-saline conditions, which was comparable to the control (Without NaCl) of K106 and Olour (**Figure 10B**). Exposure to 60 mM NaCl significantly increased MDA levels across all rootstock genotypes, with the highest value (23.74 nmol g^-1^ FW) observed in H07, indicating its greater sensitivity to salinity stress. Exogenous application of MT effectively reduced lipid peroxidation, resulting in lower MDA content. Treatment with 150 µM MT reduced MDA levels by 10.90%, 7.35%, 9.90%, 6.40%, 5.45%, and 7.56% in Kurukkan, Turpentine, Olour, K106, K109, and H07, respectively, compared to plants exposed to 60 mM NaCl alone. These results suggest that MT mitigates oxidative damage by limiting membrane lipid peroxidation.

### 3.7 Correlation analysis

**Figure 11:**
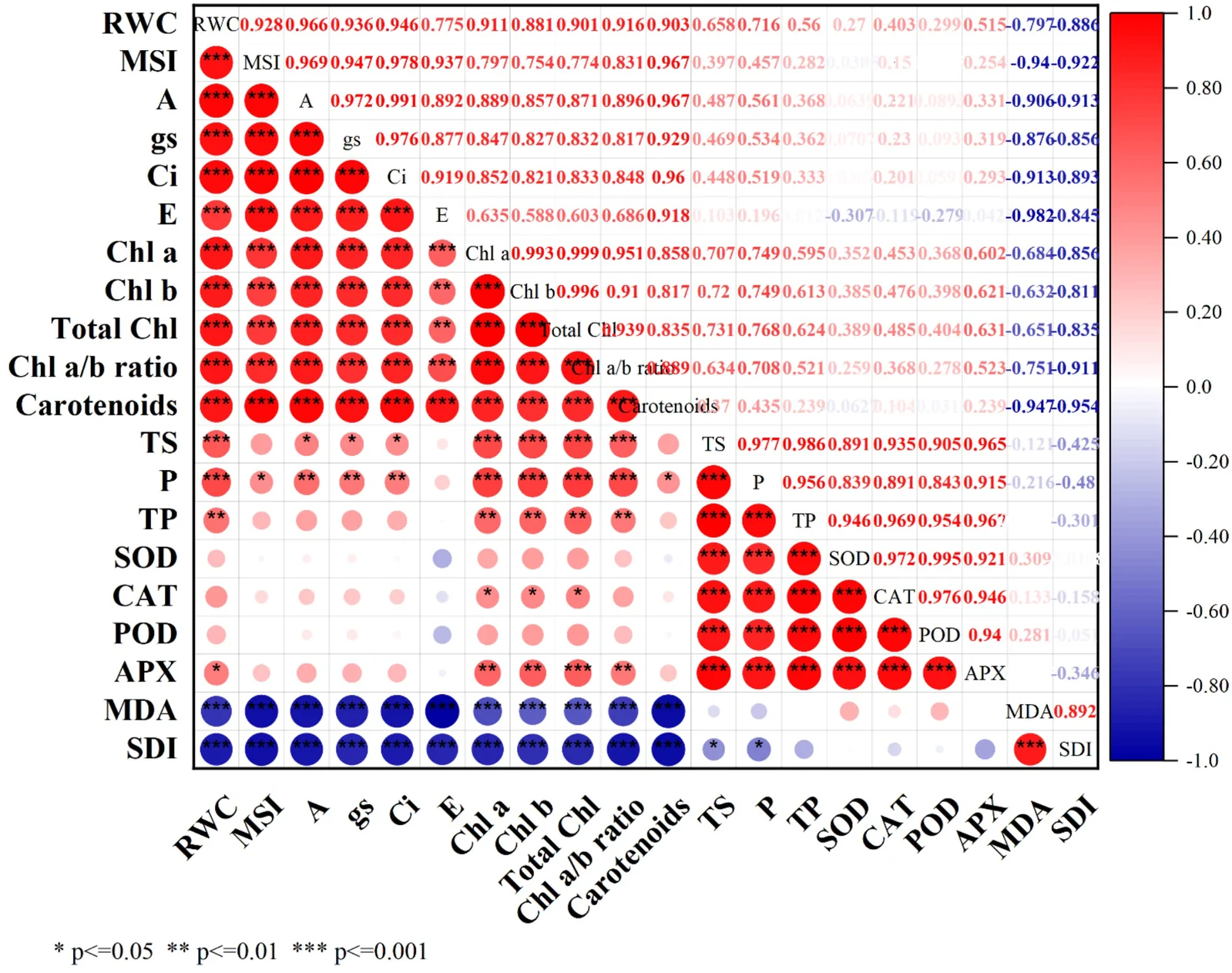
Heat map illustrating Pearson’s correlation coefficients among physiological and biochemical traits assessed using different salinity levels and melatonin concentration in various mango rootstock genotypes. Six-month-old mango seedlings of Kurukkan, Turpentine, K106, K109, H 07, and Olour were subjected to different treatment combinations.

The correlation analysis revealed strong and consistent relationships among physiological and biochemical parameters under the experimental conditions (**Figure 11**). RWC showed strongest correlation with parameters like MSI (r = 0.928; p<0.05), A (r = 0.969; p<0.05), gs (r = 0.972; p<0.05), Ci (r = 0.976; p<0.05), Chl a/b (r = 0.916; p<0.05) and negative correlation with MDA (r = -0.797; p<0.05) and SDI (r = -0.886; p<0.05). Similarly, other parameters like MSI, A, gs, E, Ci, Chlorophyll and carotenoids were positively correlated with one another but negatively associated with MDA and SDI. Total sugar exhibited strong correlation with proline (r = 0.977; p<0.05), total phenol (r = 0.986; p<0.05) and antioxidant enzymes such as APX (r = 0.965; p<0.05), CAT (r = 0.935; p<0.05) and POD (r = 0.905; p<0.05) while maintaining a negative relationship with MDA (r = -0.121; p<0.05) and SDI (r =-0.425; p<0.05). Overall, the correlation matrix clearly demonstrates that enhanced physiological status, pigment stability, osmolyte accumulation, and antioxidant activity are positively interlinked. In contrast, MDA accumulation and SDI are negatively associated with these traits, underscoring their role as indicators of stress-induced damage.

### 3.8 Principal component analysis

**Figure 12:**
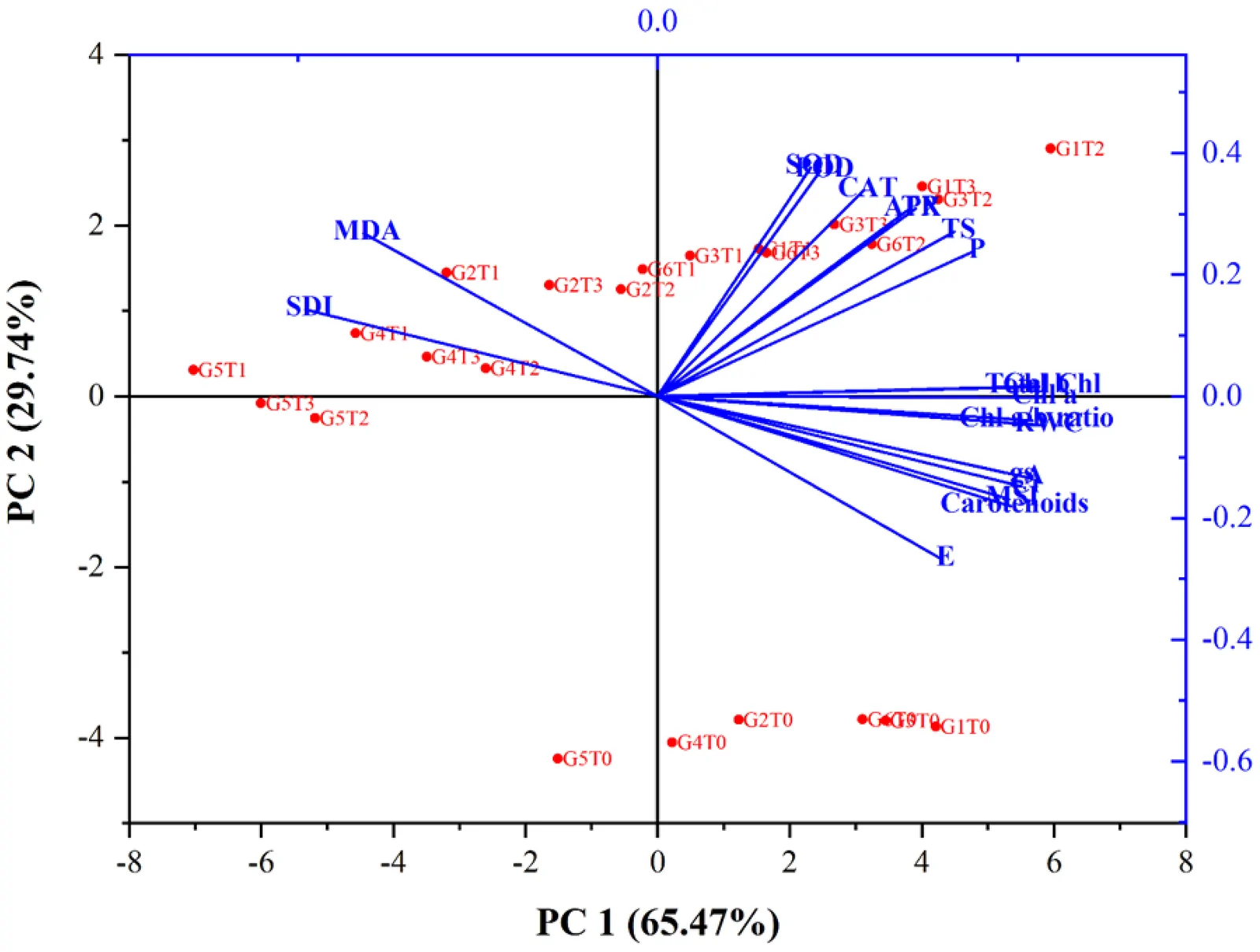
Parameters contribution and distribution of treatments on the scatter plot, along with PC1 and PC2. T0: without NaCl, T1: 60 mM NaCl, T2: 60 mM NaCl+ 150 uM MT, T3: 60 mM NaCl+ 200 uM MT, G1: Kurukkan, G2: Turpentine, G3: K 106, G4: K 109, G5: H 07, G6: Olour.

The combined effects of melatonin and salinity on six mango rootstock genotypes were assessed using Principal Component Analysis (PCA). The scree plot (**Supplementary Figure 1**) displays the eigenvalues of the principal components and illustrates their contribution to the total variance. The first two principal components (PC1 and PC2) together accounted for 95.21% of the total variation, with PC1 contributing 65.47% and PC2 contributing 29.74%. PC1 primarily separated traits associated with physiological performance and antioxidant defense (positive axis) from oxidative stress indicators (negative axis), while PC2 further distinguished among treatment-specific responses (**Figure 12**). The PCA biplot revealed a clear grouping of variables. Coordinated defensive responses were indicated by the clustering of total proteins, sugars, and antioxidant enzymes (SOD, CAT, POD, and APX). Chlorophyll concentration, carotenoids, and gas exchange parameters were among the photosynthetic characteristics that formed another cluster linked to metabolic efficiency. In contrast, MDA and SDI were positioned on the negative side of PC1, indicating their inverse relationship with most physiological and biochemical parameters. Seedlings exposed to 60 mM NaCl (T_1_) shifted towards the negative side of PC1, closely aligned with MDA and SDI, indicating increased oxidative damage. In contrast, melatonin-treated plants (T_2_ and T_3_) were positioned on the positive side of PC1, clustering with antioxidant enzymes and metabolic parameters, indicating improved stress tolerance. Genotype-specific variation was evident, with sensitive genotypes such as H 07 and K 109 shifted towards the MDA and SDI-associated region, reflecting increased lipid peroxidation and reduced stress tolerance. Tolerant genotypes like Kurukkan, Turpentine, and Olour maintained closer association with photosynthetic and metabolic traits. Somewhat tolerant genotypes, including K 106 and Turpentine, displayed intermediate reactions.

## 4 Discussion

Salt stress triggers a cascade of oxidative and osmotic disturbances that collectively disrupt cellular homeostasis. The initial osmotic imbalance restricts water uptake, while excessive accumulation of Na_+_ and Cl_-_ ions induces ionic toxicity, leading to overproduction of reactive oxygen species (ROS). This ROS burst destabilizes cellular redox equilibrium, promotes lipid peroxidation, and increases membrane permeability, ultimately impairing plant growth and development (Hasanuzzaman et al., 2021). Mangoes are India’s most popular fruit crop. Still, their extreme susceptibility to salt stress, particularly during seedling establishment, has a direct negative impact on growers’ productivity and financial stability (Dubey et al., 2007). The present study demonstrates that exogenous MT significantly mitigates these stress-induced damages through coordinated regulation of redox homeostasis, osmotic balance, and membrane stability. MT plays a crucial role as a frontline antioxidant in controlling plant growth and providing resilience to environmental extremes (Zhang et al., 2013). Here, we analyzed the physico-chemical response of six mango rootstock genotypes subjected to different levels of salinity and melatonin treatments. Our findings demonstrate that MT application markedly improves salt tolerance through coordinated physiological and biochemical modifications. Notably, the effect was more pronounced at 150 µM MT, supporting the concept of an optimal concentration range for melatonin-mediated stress mitigation. This observation supports the hormetic nature of melatonin, in which moderate doses improve physiological performance, whereas higher concentrations confer no additional benefit. However, the magnitude of this response was genotype-dependent, with tolerant genotypes showing a more pronounced improvement than susceptible genotypes.

RWC and MSI are key physiological indicators widely used to assess abiotic stress tolerance in horticultural crops. In the present study, exposure of mango rootstock genotypes to salinity stress resulted in a pronounced reduction in both RWC and MSI, indicating compromised water status and membrane damage. Salt-induced ROS accumulation typically initiates lipid peroxidation, disrupting the phospholipid bilayer and increasing electrolyte leakage. However, exogenous application of MT substantially alleviated these adverse effects, as MT-treated plants maintained significantly higher RWC and improved membrane stability compared to untreated salt-stressed plants. Maintaining a higher RWC suggests that MT might help with osmotic adjustment and/or regulate stomatal activity to reduce excessive water loss. Simultaneously, the increase in MSI suggests that MT plays a protective role in maintaining membrane integrity by reducing ROS-induced lipid peroxidation, thereby preserving cellular structure and function under stress. The results are consistent with earlier reports; for example, Zahedi et al. (2021) demonstrated improved water relations and membrane stability in olive under salinity stress following MT application. Likewise, Ali et al. (2021) observed that NaCl-induced reductions in growth, chlorophyll content, RWC, and MSI in tomato were significantly reversed by MT treatment, with notable improvements in RWC (up to 38.4%) and MSI (up to 40.7%).

Photosynthetic inhibition under salinity is primarily driven by stomatal closure and damage to the photosynthetic apparatus (Takahashi and Murata, 2008). Consistent with this, the present study recorded significant declines in gas exchange parameters, including A, gs, E, and Ci under salt stress. However, foliar application of MT, particularly at 150 μM, effectively mitigated these reductions and resulted in substantial recovery of all measured photosynthetic attributes, indicating improved photosynthetic efficiency under saline conditions (Ke et al., 2018). This response may be attributed to MT mediated hormonal crosstalk, particularly its interaction with abscisic acid (ABA), which governs stomatal dynamics under stress. By modulating ABA signaling, MT likely prevents excessive stomatal closure, thereby ensuring sustained CO_2_ assimilation and photosynthetic activity. The capacity of MT to restore photosynthetic performance has been widely reported in perennial fruit crops. Studies in *Malus* spp. have shown that MT improves Photosystem II (PSII) efficiency and alleviates drought-induced inhibition of photosynthesis, thereby enabling leaves to maintain better water status, membrane integrity, and pigment levels (Li et al., 2015). Similarly, Hu et al. (2022) demonstrated that the application of 100 μM MT mitigated NaCl-induced stress in tomato seedlings by enhancing photosynthetic efficiency, stomatal conductance, and PSII activity, and reducing leaf injury. In the present study, the observed increases in transpiration rate and stomatal opening further suggest that MT may function as a regulatory signal to prevent excessive stomatal closure under stress conditions. This result aligns with the findings of Zhao et al. (2021), who reported that 100 μM melatonin application enhanced stomatal conductance in maize under drought stress, thereby maintaining photosynthetic activity under stressful environmental conditions.

Salinity stress severely compromised the integrity of the light-harvesting complex, leading to the depletion of chlorophyll and carotenoid concentrations. Melatonin can inhibit chlorophyll degradation under abiotic stress, increase leaf photosynthetic activity, and promote the accumulation of plant dry matter (Yang et al., 2018). The exogenous application of 150 μM and 200 μM MT effectively safeguarded these pigments, as evidenced by significantly higher retention rates of Chl a, Chl b, and carotenoids. These findings are consistent with earlier reports on pistachio (Kamiab, 2020) and blueberry (Wenfei et al., 2023) under salinity stress. Salinity-induced tissue damage varied among genotypes, reflecting differences in their sensitivity to salt stress. The highest degree of tissue necrosis was observed in sensitive genotypes such as K109 and H07 under 60 mM NaCl treatment. This may be attributed to excessive accumulation of chloride ions (Cl_-_) in leaf tissues, which likely disrupted water and nutrient uptake, leading to enhanced generation of reactive oxygen species (ROS). The resulting oxidative stress contributes to chlorophyll degradation and reduced ATP synthesis, as reported by Yang et al. (2011) and Shankar et al. (2023). Melatonin application mitigated these adverse effects by enhancing antioxidant enzyme activity and promoting efficient ROS scavenging, thereby reducing tissue damage and supporting the maintenance of cellular integrity and new growth.

Under saline conditions, antioxidant enzymes serve as a primary defense by sequestering reactive peroxides and converting them into non-toxic metabolites, effectively buffering the cellular environment against oxidative stress. Our findings demonstrate that MT supplementation significantly fortifies this enzymatic framework in mango seedlings exposed to 60 mM salinity by upregulating the expression of antioxidant enzymes. CAT, SOD, POD and APX. It acts as a potent redox regulator, restoring cellular homeostasis by modulating the fine balance between ROS production and scavenging, thereby reinforcing membrane structural integrity (Manchester et al., 2015). This observation aligns with recent evidence in *Punica granatum*, where 150 μM MT enhances the activities of SOD, POD, and CAT by 14.3%, 21.7%, and 11.7%, respectively, thereby markedly stabilizing the phospholipid bilayer (Yang et al., 2025). Similar physiological resilience was observed in olive seedlings, in which MT application up-regulated the APX and CAT pathways, leading to concurrent reductions in electrolyte leakage and peroxidative damage (Zahedi et al., 2021).

Salinity stress led to an increased accumulation of osmomodulating compounds, including proline, total sugar, and phenols, which play a crucial role in alleviating stress effects by lowering cellular water potential and maintaining turgor. In addition to their osmotic functions, these metabolites act as non-enzymatic antioxidants, directly scavenging reactive oxygen species (ROS) and buffering oxidative stress, thereby complementing the enzymatic antioxidant system. The increase in total phenol content under melatonin treatment is physiologically significant, as phenolic compounds act as important non-enzymatic antioxidants that scavenge reactive oxygen species generated under salt stress. By mitigating oxidative damage, these compounds contribute to membrane stabilization, reduced cellular injury, and enhanced stress tolerance in mango rootstocks. This interpretation is further supported by the findings of Perveen et al. (2024), who demonstrated a strong association between phenolic profiles and salinity tolerance in mango, emphasizing the pivotal role of phenolic metabolism in the physiological mechanisms underlying salt stress adaptation. Exogenous MT application further increased metabolite levels, thereby reducing salinity-induced damage. Liu et al. (2018) observed that MT treatment increased proline levels by 26.8% in salt-stressed rapeseed seedlings, aiding in osmotic regulation. According to Liang et al. (2019), in kiwi seedlings under drought stress, melatonin at 100 μM reduced lipid peroxidation and pigment breakdown, thereby reducing cell membrane damage and encouraging the buildup of osmolytes. In addition, MT has been reported to improve fruit quality and delay senescence by maintaining elevated levels of ascorbic acid, total phenolics, and flavonoids, as evidenced by Cai et al. (2024). The present findings are in agreement with previous reports demonstrating the protective effects of melatonin in drought-stressed banana and other fruit crops (Hassan et al., 2022).

The PCA biplot provides clear evidence that the balance between oxidative damage and the activation of defense and physiological mechanisms primarily governs salinity stress in mango rootstocks. The inverse association between MDA and photosynthetic characteristics, as well as antioxidant enzymes, emphasizes how important oxidative stress is in restricting plant performance in saline environments. By strengthening antioxidant defense systems, melatonin treatment reduced the adverse effects of salt. The clustering of MT treated samples with SOD, CAT, POD, and APX shows enhanced metabolic activity, membrane stability, and ROS detoxification (Khobragade et al., 2025). The more pronounced effect observed at 150 µM MT supports the concept of an optimal concentration range for melatonin action. This concentration likely maximized antioxidant defense without disrupting cellular signaling, whereas higher doses were not as effective. There were clear genotypic differences: Kurukkan, K 106, and Olour demonstrated significant tolerance through efficient antioxidant activation, whereas K 109 and H 07 remained vulnerable. This genotype dependent response may be linked to the inherent differences in antioxidant capacity, ion transport efficiency, and signaling networks. Tolerant genotypes exhibited a more robust activation of redox and osmotic defense mechanisms, suggesting that MT acts synergistically with intrinsic genetic traits to enhance stress resilience.

Overall, the findings show that melatonin at an optimal dose improves salinity tolerance by changing the ratio of physiological functions to oxidative defense, with genotype being a key factor in determining responsiveness. Despite the limited visible recovery, MT treatment led to significant improvements in key physiological and biochemical attributes, including relative water content, membrane stability index, antioxidant enzyme activities, and osmolyte accumulation. These responses indicate that melatonin primarily confers stress tolerance at the cellular and metabolic levels, effects that may not be immediately evident at the morphological level, particularly under severe stress or within a short experimental duration.

## 5 Conclusion

Mango, a salt-sensitive crop, exhibits pronounced declines in growth and development under saline conditions, with severe stress often leading to plant mortality. The present study demonstrates that exogenous melatonin (MT) effectively alleviates salinity-induced damage in mango seedlings by improving key physiological and biochemical parameters across different rootstock genotypes. These improvements contribute to enhanced stress tolerance and support overall plant survival under saline conditions. Although the extent of improvement varied with genotype and MT concentration, application at 150 μM consistently produced the most beneficial outcomes. This enhancement in salt tolerance appears to result from the coordinated activation of multiple protective mechanisms. Overall, the findings highlight the potential of 150 μM MT as a practical strategy to strengthen mango rootstocks against salinity stress, thereby supporting sustainable production in salt-affected areas. Further findings will be validated using composite plants (rootstock + scion) with the same MT concentrations.

## Data Availability

The original contributions presented in the study are included in the article/**Supplementary Material**. Further inquiries can be directed to the corresponding author.
