# Supplementary material for: Melatonin-mediated alleviation of salinity stress in diverse mango (Mangifera indica L.) rootstock genotypes
Source: Front Plant Sci. 2026 Aug 26;17:1873066. doi: 10.3389/fpls.2026.1873066 (PMC13553784; doi:10.3389/fpls.2026.1873066)
Supplement: Supplementary file 1 [file Table1.docx]

Supplementary Figure S1: A scree plot illustrating the factors investigated during the experimentation.
